# Anatomical Risk Patterns for Patellofemoral Instability in Skeletally Immature Patients: A Sex-Stratified MRI Study

**DOI:** 10.3390/jcm14155519

**Published:** 2025-08-05

**Authors:** René Schroedter, Amir Koutp, Bernhard Guggenberger, Martin Svehlik, Sebastian Tschauner, Tanja Kraus

**Affiliations:** 1 Pediatric and Adolescent Unit, Department of Trauma and Orthopedics, Medical University of Graz, 8036 Graz, Austria; rene.schroedter@medunigraz.at (R.S.);; 2 Department of Trauma and Orthopedics, Medical University of Graz, 8036 Graz, Austria; 3 Medical University of Graz, 8036 Graz, Austria; 4 Division of Paediatric Radiology, Department of Radiology, Medical University of Graz, 8036 Graz, Austria

**Keywords:** trauma orthopedic, lateral patellar dislocation, knee injury, pediatric orthopedic, orthopedic surgery

## Abstract

**Background/Objectives:** Lateral patellar dislocation (LPD) is a common pathology of the adolescent knee and a major predisposing factor for patellofemoral instability (PFI). The pathogenesis of PFI involves a combination of anatomical and biomechanical contributors, with increasing evidence pointing to sex-specific differences in knee morphology. Despite this, the developmental course of these parameters and their variation between sexes remain insufficiently characterized. This study aims to investigate sex-related differences in patellofemoral joint geometry among skeletally immature patients with a history of PFI, focusing on how these anatomical variations evolve with increasing knee size, as represented by femoral condylar width. **Methods:** A total of 315 knee MRIs from patients under 18 years with documented PFI were retrospectively analyzed. Trochlear morphology, patellar tilt, axial positioning, and sagittal alignment were assessed using established MRI-based parameters. All measurements were normalized to bicondylar width to account for individual knee size. Sex-specific comparisons were performed using independent *t*-tests and linear regression analysis. **Results:** Females exhibited significantly smaller femoral widths, shallower trochlear depth (TD), shorter tibial tubercle–posterior cruciate ligament (TTPCL) distances, and lower patellar trochlear index (PTI) values compared to males (*p* < 0.05). In males, increasing femoral width was associated with progressive normalization of patellar tilt and sagittal alignment parameters. In contrast, these alignment parameters in females remained largely unchanged or worsened across different femoral sizes. Additionally, patellar inclination angle and PTI were significantly influenced by knee size in males (*p* < 0.05), whereas no such relationship was identified in females. **Conclusions**: Sex-specific morphological differences in patellofemoral geometry are evident early in development and evolve distinctly with growth. These differences may contribute to the higher prevalence of PFI in females and underscore the importance of considering sex and knee size in anatomical assessments.

## 1. Introduction

Lateral patella dislocation (LPD) is a common patellofemoral joint pathology in adolescents, with an incidence ranging from 6 to 50 per 100,000 individuals aged 10 to 17, peaking around age 15 [1,2,3]. Nearly half of those affected develop patellofemoral osteoarthritis within 25 years of the initial dislocation [4].

Multiple anatomical and biomechanical factors contribute to patellofemoral instability, including trochlear dysplasia, patella alta, increased patellar tilt, lateralized patella (e.g., elevated TT-TG/TT-PCL distance), and valgus or rotational malalignment [5,6,7,8,9,10]. In addition, high-risk dynamic movement patterns—such as valgus collapse during pivoting or twisting motions—can further exacerbate instability [2,5,11,12,13,14,15,16,17,18].

Importantly, sex-based differences significantly influence the for lateral patella dislocation and presentation of those patients. Females demonstrate a 33% higher risk of recurrent LPD when compared to males [19,20,21], a disparity attributed to both morphological and biomechanical factors. These include greater physiological valgus alignment, increased ligamentous laxity, and differences in neuromuscular control. Additionally, several studies have highlighted sex-specific geometric characteristics of the knee joint that may underlie this increased risk. For example, variations in trochlear depth and sulcus angle, patellar height and tilt, femoral condylar morphology, and tibial tubercle lateralization have all been shown to differ systematically between males and females [22,23,24,25,26]. Distinct differences between males and females in patellofemoral anatomy—such as trochlear depth, sulcus angle, patellar height and tilt, femoral condylar shape, and tibial tubercle lateralization—have been well established and are already present during skeletal growth.

These geometric differences are not only relevant for understanding injury susceptibility but also constitute fundamental anatomical knowledge with implications for joint function and mechanical loading across developmental stages. They may reflect sexually dysmorphic growth trajectories influenced by hormonal, genetic, and biomechanical factors during adolescence—a period marked by rapid change in musculoskeletal structure and coordination [23,27,28]. Understanding and integrating these sex-based anatomical variations is increasingly recognized as essential—not only for refining clinical assessment and research models but also as part of a comprehensive anatomical framework for knee joint evaluation. As Hirschmann et al. [29] have already emphasized, individualized and anatomically precise assessments are a cornerstone of modern orthopedic research.

While chronological age remains an important consideration in assessing patellofemoral instability, it is often an imprecise proxy for skeletal maturity. Adolescents of the same age may exhibit substantial variability in body height, femoral morphology, and joint dimensions [6,7,30,31,32,33]. Shelbourne et al. [34] demonstrated a strong correlation between femoral width at the condylar level and body height, suggesting that morphometric parameters may serve as more reliable markers of knee development.

The present study aims to characterize sex-specific morphological variations in the patellofemoral joint in skeletally immature patients with a history of lateral patella dislocation. Through a detailed analysis of geometric parameters—including trochlear morphology, patellar alignment, and femoral-tibial relationships—this work seeks to identify consistent patterns associated with biological sex. By establishing a clearer anatomical basis for these differences, the findings may contribute to more nuanced interpretations of risk profiles during critical periods of musculoskeletal development.

## 2. Materials and Methods

This study analyzed magnetic resonance imaging (MRI) scans of growing patients treated for patellofemoral instability (PFI) with at least one non-traumatic lateral patella dislocation at our institution, with a focus on gender-related differences. MRI data were retrieved from the local PACS (Picture Archiving and Communication System) server. The study received approval from the institutional review board (EK Nr. 34-119 ex 21/22), and due to its retrospective design, informed consent was not required.

Patients under the age of 18 who had experienced at least one, non-traumatic lateral patella dislocation were included for this work. Exclusion criteria for both groups were unclear clinical history, incomplete records, osteochondral lesions, other bony knee injuries (such as tibial plateau or femoral condyle fractures), and soft tissue injuries unrelated to the patellofemoral joint (e.g., cruciate ligament, meniscal, or collateral ligament injuries). Additionally, patients with congenital, skeletal, or neurological disorders, as well as those with a history of prior knee surgery, were excluded. Data collection spanned from 2000 to 2022.

A total of 648 knee MRI scans from patients with a history of PFI were identified, of which 315 met the inclusion criteria. Anatomical knee measurements relevant to PFI were obtained and compared between male and female patients, with adjustments made for individual knee size. Patient medical histories were reviewed through clinical reports and MRI referral diagnoses. In the PFI group, at least one documented patellar dislocation was required, and only the first MRI following the diagnosis was included in the analysis.

### 2.1. MRI Measurements

To identify pathological parameters associated with an increased risk of non-traumatic lateral patella dislocation, MRI scans were systematically analyzed. Measurements were conducted by two certified orthopedic surgeons with over 15 years of experience and two orthopedic residents with more than 5 years of clinical training. The reliability of these measurements was supported by the previously validated literature [35].

A key focus was trochlear dysplasia, a critical factor in PFI, which was assessed using three quantitative metrics: trochlear depth (TD), trochlear sulcus angle (TSA), and lateral trochlear inclination angle (LTIA) [14,18]. These parameters were selected for their reliability in characterizing trochlear morphology and its role in dysplasia.

Patellar tilt was quantified using three angular measurements: the Angle of Fulkerson (FULK), the Angle of Laurin (LAUR), and the patellar inclination angle (TILT) [12]. Additionally, patellar lateralization in the axial plane was evaluated through tibial tuberosity to trochlear groove distance (TTTG) and tibial tuberosity to posterior cruciate ligament distance (TTPCL) [2,14].

Sagittal patellofemoral alignment, which plays a crucial role in PFI by influencing the dynamic interaction between the patella and trochlea during knee motion, was assessed using the Patellotrochlear Index (PTI) and the Caton-Deschamps Index (CDI). These indices were chosen for their established reliability in skeletally immature patients [11,15].

To provide an individualized perspective on knee geometry, measurements were normalized relative to the bicondylar width, defined as the widest point of the femur on axial slices. This approach aimed to enhance the accuracy of anatomical assessments in pediatric patients.

A detailed overview of all measurement parameters is available in the supplementary figures (Supplementary Materials).

All measurements were conducted using the MicroDicom DICOM (Digital Imaging and Communications in Medicine) viewer (MicroDicom Ltd., Sofia, Bulgaria). Demographic data extracted from the DICOM files included patient age at the time of MRI, examination date, and laterality.

### 2.2. Statistical Analysis

The statistical analysis was performed using SPSS Version 28.0 (IBM, Armonk, NY, USA). Data distribution was assessed using Q-Q plots, and all values were assumed to follow a normal distribution. To show differences in knee size between females and males, an independent samples *t*-test was applied for femoral width in the age groups ≤11, 12–14 and ≥15 years (prepubescent, pubertal and late puberty). Furthermore, correlation analysis (Pearson) was performed for age and femoral width in each of the two groups to screen for a statistically significant correlation between those parameters. For all other parameters, an independent samples *t*-test was used to determine whether significant differences existed in the measurements of anatomic risk factors between the female and male group. Variability in the data was represented by the standard error of the mean (SEM). Statistical significance for the testing was set at *p* < 0.05.

To assess the relationship between knee size and patellofemoral geometry, a linear regression analysis was conducted separately for the male and female group. Femoral width was treated as a continuous independent variable, while all patellofemoral geometry measurements were treated as continuous dependent variables. Additionally, a one-way ANOVA was performed within each group (female and male) to identify significant differences in these measurements in relation to knee size. Statistical significance was defined as *p* < 0.05.

## 3. Results

### 3.1. Patient Demographics

A total of 315 knees with a documented history of at least one time non-traumatic lateral patella dislocation were analyzed in this study (179 left knees and 136 right knees). The female group consisted of 169 patients while the male group included 146 (Table 1). Pearson correlation analysis revealed coefficients of 0.213 in the female group and 0.241 in the male group when testing for age and femoral width.

The mean age was 14.3 years for females and 14.7 years for males, with an overall age range of 8 to 18 years. The mean femoral width across the age groups (≤11, 12–14, ≥15 years) differed significantly between females and males, with all comparisons showing statistical significance (*p* < 0.05) (Table 1).

When comparing group means (Table 2), significant differences were found for TD, TTPCL, and PTI (*p* < 0.05). None of the other measured parameters showed meaningful differences between the groups (*p* > 0.05).

### 3.2. Trochlea Dysplasia

**Figure 1:**
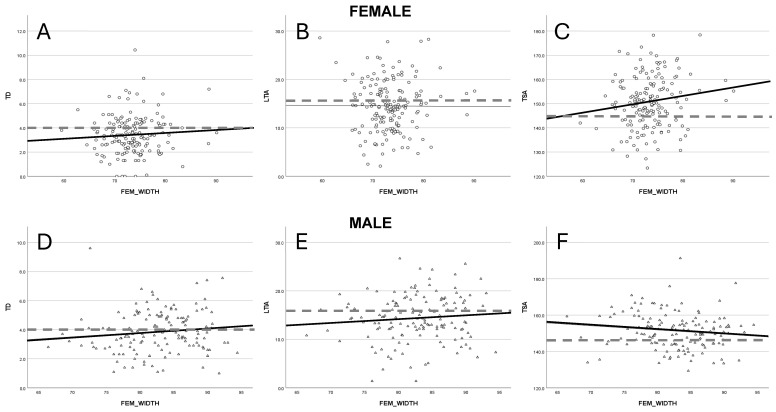
Illustrates the changes for trochlear dysplasia, represented by trochlear depth [TD] (**A**,**D**), lateral trochlear inclination angle [LTIA] (**B**,**E**) and the trochlear sulcus angle [TSA] (**C**,**F**) with respect to gender. The dashed lines are marking the cut off for abnormal values for TD (<4 mm), LTIA (<17°) and TSA (<145°).

An association between greater femoral width and increased trochlear depth was identified in both male (R^2^ = 0.006) and female (R^2^ = −0.001) cohorts (Figure 1A). In contrast, divergent patterns emerged for the trochlear sulcus angle (TSA): a reduction was observed in males (R^2^ = 0.01), while females (R^2^ = 0.014) exhibited a slight increase (Figure 1B). The lateral trochlear inclination angle (LTIA) showed a mild upward trend in males (R^2^ = 0.007) as femoral width increased, whereas the values remained stable in females (R^2^ = 0.001) (Figure 1C). However, none of these trochlear dysplasia parameters reached statistical significance, as indicated by linear regression analysis (*p* > 0.05 for all comparisons).

### 3.3. Patellar Tilt

**Figure 2:**
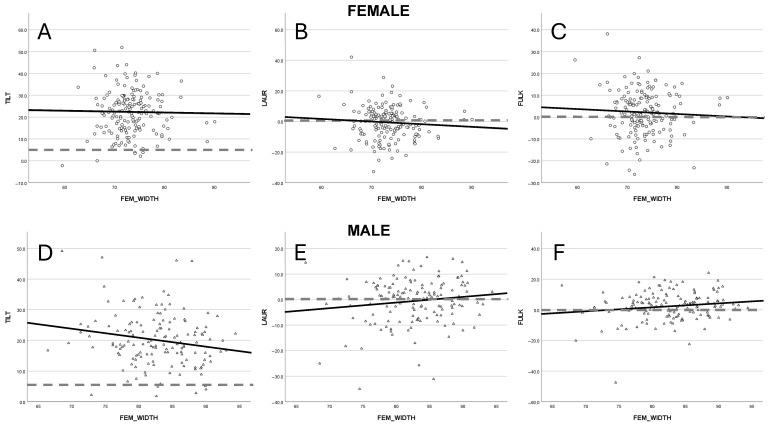
Illustrates the changes for patellar tilt, represented by the patellar inclination angle [TILT] (**A**,**D**), Angle of Laurin [LAUR] (**B**,**E**) and the Angle of Fulkerson [FULK] (**C**,**F**) with respect to gender. The dashed lines are marking the cut off for abnormal values for TILT (>5°), LAUR (<0°) and FULK (<0°).

Patellar tilt was evaluated using the Angle of Fulkerson, Angle of Laurin and the patellar inclination angle (TILT). Both the Fulkerson and Laurin angles demonstrated a trend toward higher values in males (FULK R^2^ = 0.021, LAUR R^2^ = 0.019) and a reduction in females (FULK R^2^ = 0.003, LAUR R^2^ = 0.007) with increasing femoral width (Figure 2A,B). Nevertheless, these tendencies meagerly did not achieve statistical significance in the regression model. The TILT showed a statistically significant reduction of 2.7° per 10 mm increase in femoral width in the male cohort (*p* = 0.038, R^2^ = 0.03), while no appreciable change was noted among females (R^2^ = 0.001) (Figure 2C).

### 3.4. Axial Patellar Positioning

**Figure 3:**
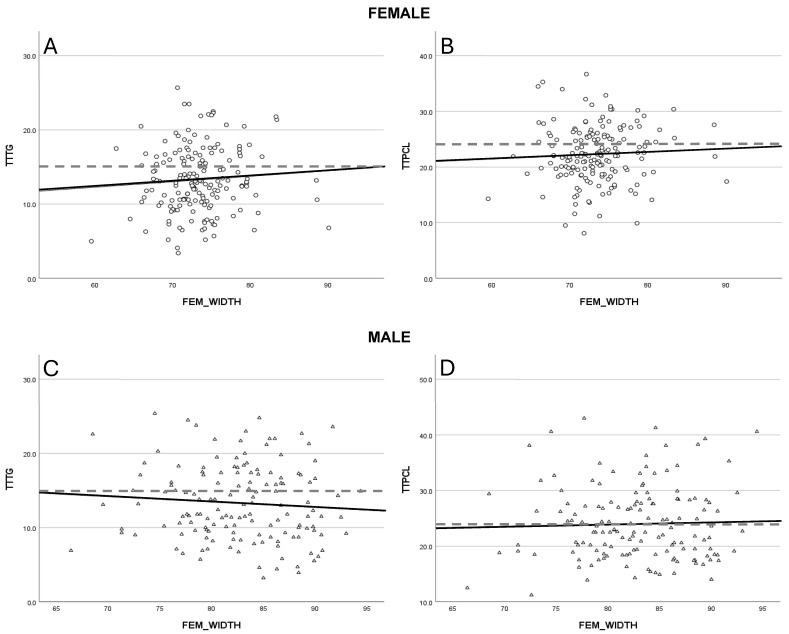
Illustrates the changes for lateralization of the patella, represented by the tibial tubercle to trochlear groove distance [TTTG] (**A**,**C**) and the tibial tubercle to posterior cruciate ligament distance [TTPCL] (**B**,**D**) with respect to gender. The dashed lines are marking the cut off for abnormal values for TTTG (>15 mm) and TTPCL (>24 mm).

The tibial tubercle–posterior cruciate ligament (TTPCL) distance exhibited a minor increase in males (R^2^ = 0.002) and females (R^2^ = 0.003), whereas the tibial tubercle–trochlear groove (TTTG) distance increased in females (R^2^ = 0.005) but slightly declined in males (R^2^ = 0.005) with greater femoral width (Figure 3A,B). Despite these trends, statistical significance was not achieved in the linear regression analysis.

### 3.5. Sagittal Patellofemoral Alignment

**Figure 4:**
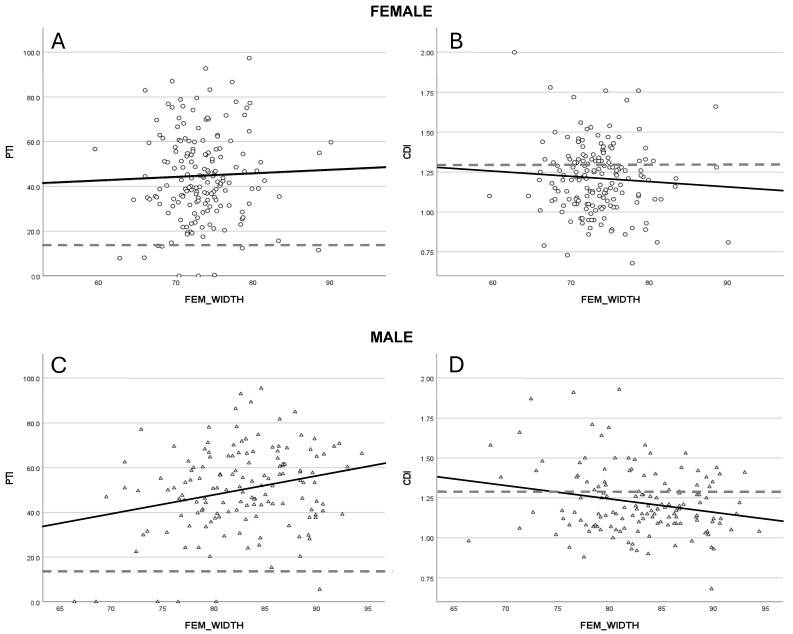
Illustrates the changes in patellar height, represented by the Patellotrochlear Index [PTI] (**A**,**C**) and the Caton-Dechamps index [CDI] (**B**,**D**) with respect to gender. The dashed lines are marking the cut off for abnormal values for PTI (<12.5) and CDI (>1.3).

A significant increase of 8.6% in the patellotrochlear index (PTI) was observed in male patients for every 10 mm increment in femoral width (*p* = 0.003, R^2^ = 0.062), whereas only a very modest increase was found in the female (R^2^ = 0.002) group (Figure 4A). Additionally, the Caton–Deschamps Index (CDI) demonstrated a significant reduction of 0.08 per 10 mm increase in femoral width among males (*p* = 0.008, R^2^ = 0.05), with females (R^2^ = 0.005) showing a less pronounced decline (Figure 4B).

## 4. Discussion

This study aimed to investigate gender-specific anatomical risk factors for patellofemoral instability (PFI) in children and adolescents, focusing particularly on how these parameters evolve with increasing femoral width. Femoral width was selected over chronological age as the primary variable due to significant discrepancies that can exist between biological and chronological maturation, along with the well-established correlation between femoral width and body height [6,30,31,32,34]. This rationale is further supported by our data, which demonstrates a weak correlation with coefficients of 0.213 in the female and 0.241 in the male group between femoral width and chronological age in both sexes.

Significant differences in mean values were observed for femoral width, trochlear depth (TD), tibial tubercle–posterior cruciate ligament distance (TTPCL), and the patellotrochlear index (PTI). Female patients, on average, exhibited approximately 10 mm less femoral width than their male counterparts within the same age category. These size-related disparities provide a plausible explanation for the observed differences in TD, TTPCL, and PTI, where knee dimensions play a substantial role in determining measurement outcomes.

Statistically significant changes were identified in patellar inclination angle as well as in sagittal alignment parameters, specifically PTI and the Caton-Deschamps Index (CDI), in relation to femoral width. Additionally, most evaluated variables displayed clear trends toward change with respect to their individual gender. In general, male patients exhibited a tendency toward normalization of values—shifting toward more physiologic ranges—while female patients showed values that are considered to be in a pathological range. These findings support the hypothesis that risk factors for PFI begin to manifest early in musculoskeletal development and progress in a gender-specific manner during growth [25,36].

The parameters TD, LTIA, and TSA are widely recognized as critical indicators of trochlear morphology and dysplasia [14,18]. Decreased TD and LTIA values have been associated with higher PFI risk, while elevated TSA values reflect a flatter, more dysplastic trochlea. Gender-specific differences appear to play a role, with trochlear dysplasia reported more frequently in female patients [24]. Orellana et al. [36] demonstrated progressive worsening of trochlear morphology over time when comparing successive measurements in patients with PFI to normally developing pediatric controls. Similarly, Pruneski et al. [25] reported normalization of parameters over time in knees without PFI, suggesting that failure to follow this normative trajectory may indicate early developmental aberrations contributing to instability. Moreover, Parikh et al. [37] stated that there might be a genetic predisposition to trochlea dysplasia. Results show slightly rising values for TD in both genders with women having a shallower trochlea in TSA and LTIA compared to the male cohort. This matches with Pagliazzi et al. [38] who described similar changes in trochlear development like increasing lateral condyle height leading to a potentially higher trochlea depth values but females having a flatter trochlea over the course of growth.

The patellar inclination angle, Fulkerson angle, and Laurin angle all showed gender-divergent trends. Male patients demonstrated a tendency toward decreased patellar tilt with increasing femoral width—approaching normative values—whereas females maintained elevated tilt values. This trend may be linked to a greater prevalence of severe trochlear dysplasia in females [24]. Findings by Pruneski et al. [25] align with our observations, indicating a more substantial reduction in patellar tilt among males over time.

The TTPCL distance showed a mild increase in both male and female cohorts. Conversely, the TTTG increased in females but declined slightly in males. The literature on TTTG values presents mixed findings, with some studies suggesting comparable means between genders, consistent with our observations. However, a higher TTTG in females with PFI has also been reported, which may account for the increasing trend we observed in this subgroup [37]. Although not statistically significant, our results diverge from those reported by Pruneski et al. [25], indicating sex-specific patterns in the response of TTTG to growth. Furthermore, Dong et al. [39] noted that TTTG measurements exhibit reduced interrater reliability in cases with moderate to severe trochlear dysplasia. Given that approximately one-third of our cohort had at least mild dysplasia when lateral patellar dislocation occurred, this could explain discrepancies from normative developmental cohorts such as that studied by Pruneski et al. [25]. Moreover, since TTTG is influenced by femoral rotation and relies on accurate cross-joint measurements, variation in MRI acquisition protocols can affect its reliability, especially when compared across institutions or with CT data [40]. In contrast, TTPCL may serve as a more consistent indicator of tubercle lateralization, being independent of trochlear morphology.

Patellar height was assessed using both the PTI and CDI, revealing size- and gender-related variations (Figure 4A,B). In male patients, a statistically significant reduction in patellar height was observed with increasing femoral width, more pronounced than in the female cohort. Pringle et al. [24] reported higher average patellar height in males, consistent with our findings, particularly regarding the PTI. On the other hand, Pruneski et al. [25] found contrary results, with higher patellar height in females and a more marked decrease over time. This discrepancy may reflect differences in study populations, as our sample included patients with established PFI, while Pruneski et al. [25] focused on a non-PFI pediatric cohort.

This study has several limitations, chiefly its retrospective design, which may introduce selection bias, such as incomplete anthropometric data or gender distribution within the study population deviating from the literature. Prior studies, such as that by Shelbourne et al. [34], support the strong correlation between femoral width and stature, validating our approach. Nonetheless, the absence of patients under 8 years of age restricts the applicability of findings to the earliest phases of skeletal development. Furthermore, no control group was incorporated in this study to show differences in relation to non PFI patients. The lack of longitudinal follow-up limits our ability to infer growth trajectories directly, as data reflect only a single time point per patient. Being a single-center study further limits generalizability. Moreover, although the anatomical parameters were thoroughly analyzed, functional and biomechanical correlates of instability were not assessed, which could provide additional insight into clinical outcomes. Notably, the majority of cases involved adolescents nearing growth plate closure, a phase characterized by limited remaining skeletal change.

Patellofemoral instability (PFI) remains a significant clinical concern, particularly in female adolescents, due to dynamic anatomical changes during growth that complicate both diagnosis and management. Anatomical predisposition plays a central role in the etiology of PFI [33]. Our findings align with previous research highlighting gender-specific morphological risk factors and the developmental changes in joint anatomy throughout adolescence [24,25,37,38]. While risk factors in male patients tend to normalize with increasing femoral width, female patients exhibit changes that may necessitate close monitoring or early conservative interventions, such as physiotherapy or the avoidance of high-risk sports. Preventive programs targeting ACL injuries have already shown to reduce incidence rates among high-risk athletes [41]. Additionally, with regard to coronal lower limb alignment, females typically exhibit increased hip internal rotation [23,42]. Recent studies have further demonstrated that elevated hip internal rotation is associated with greater dynamic valgus, especially in comparison to males [43,44]. The increasing focus on individualized treatment approaches reinforces the importance of considering these sex-specific anatomical differences, particularly in skeletally immature patients [29].

This study reinforces the notion that anatomical risk factors for PFI evolve differently in male and female patients as femoral width increases. By integrating this work’s findings with the current literature, it proposes a framework that highlights the gender-specific developmental aspects of PFI. Future longitudinal studies following individual patients across growth stages will be essential to elucidate the full impact of sex and non-traumatic lateral patella dislocation on knee development, ultimately informing tailored treatment approaches for patients at risk of recurrent dislocation.

## Figures and Tables

**Table 1:** Shows the demographic data available for both cohorts.

Measurement		Female	Male
Group size		169	146
Age		14.3 years	14.7 years
Femoral width	Mean all	73.3 mm	82.7 mm
	Mean ≤ 11	71.9 mm	79.5 mm
	Mean 12–14	72.9 mm	81.6 mm
	Mean ≥ 15	73.9 mm	83.7 mm

**Table 2:** Shows mean, standard error of the mean as well as the results of independent *t*-test analysis for a difference between the groups. (PFI = patellofemoral instability, CON = control, SEM = Standard error of the mean, TD = trochlea depth, LTIA = lateral trochlea inclination angle, TSA = trochlea sulcus angle, LAUR = angle of Laurin, FULK = angle of Fulkerson, TILT = patella inclination angle, TTTG = tibial tubercule to trochlea groove distance, TTPCL = tibial tubercule to posterior cruciate ligament distance, PTI = Patellotrochlear-Index, CDI = Caton-Dechamps-Index).

Measurement	Male (Mean ± SEM)	Female (Mean ± SEM)	*p*-Value
TD	3.9 (±0.34)	3.4 (±0.45)	<0.05 *
LTIA	14.3 (±0.12)	14.4 (±0.12)	0.770
TSA	151.2 (±0.83)	150.7 (±0.82)	0.642
LAUR	−0.6 (±0.73)	−0.8 (±0.81)	0.818
FULK	2.4 (±0.77)	2.1 (±0.77)	0.757
TILT	20.1 (±0.71)	22.1 (±0.76)	0.064
TTTG	13.2 (±0.34)	13.3 (±0.42)	0.965
TTPCL	24.1 (±0.55)	22.3 (±0.39)	<0.05 *
PTI	44.5 (±1.57)	50.1 (±1.49)	<0.05 *
CDI	1.2 (±0.02)	1.2 (±0.02)	0.640

* *p*-values that reached statistical significance (*p* < 0.05).

## Data Availability

The data will be made available by the first author upon request.

## Supplementary Materials

The following supporting information can be downloaded at https://www.mdpi.com/article/10.3390/jcm14155519/s1, Figure S1: (A) The trochlear sulcus angle measures the angle between the medial and lateral facets of the trochlear groove; (B) Trochlear depth is calculated as the average height of the medial (a) and lateral facet (b) minus the depth of the trochlear groove (c) [(a+b)/2-c]; C) Lateral trochlear inclination is the angle between a tangential line along the posterior femoral condyles (e) and the lateral facet of the trochlear groove (d); D) Femoral width is the maximum distance between the femoral epicondyles; Figure S2: (A) The Fulkerson angle is defined as the angle between a tangential line along the posterior femoral condyles (a) and the lateral facet of the patella (b); (B) The Laurin angle is measured between the anterior condyles (c) and the lateral patellar facet (b); (C) The patellar tilt angle is the angle between a line along the posterior femoral condyles (a) and the largest axial diameter of the patella (d); Figure S3 (A) The TTTG distance is measured as the distance (d) between two perpendicular lines drawn from a tangential line to the posterior condyles (a) to the trochlear groove (c) and the midpoint of the tibial tubercle (b); (B) The TTPCL distance is determined by the distance (d) between two perpendicular lines extending from the posterior condyles (a) to the medial border of the PCL at its tibial insertion (c) and the midpoint of the tibial tubercle (b); Figure S4: The Caton-Deschamps Index (A/B) is the ratio of the distance from the anterior edge of the tibial plateau to the apex of the patella (A) divided by the length from the base to the apex of the patella (B); Figure S5 The Patellotrochlear Index is the percentage of the femoral trochlea cartilage covering the cartilage of the Patella. The length of the cartilage of the femoral trochlea (a) is divided by the length of the cartilage of the Patella (b) multiplied by 100 in a sagittal view [(a/b)*100]; The sequence with the biggest diameter of the patella is to be picked for this measurement.
