# Supplementary material for: Anatomical Risk Patterns for Patellofemoral Instability in Skeletally Immature Patients: A Sex-Stratified MRI Study
Source: J Clin Med. 2025 Aug 5;14(15):5519. doi: 10.3390/jcm14155519 (PMC12347535; doi:10.3390/jcm14155519)
Supplement: Supplementary file 1 [file jcm-14-05519-s001.zip › jcm-3709216-supplementary.pdf]

Supplementary Figures

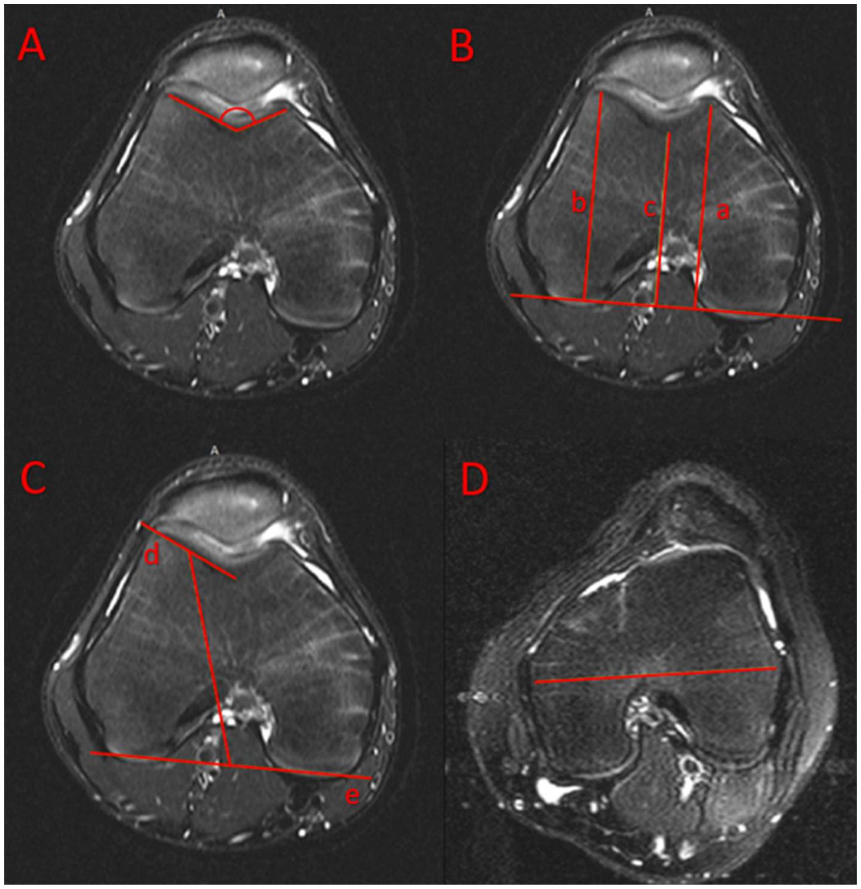


**Figure S1** A) The trochlear sulcus angle measures the angle between the medial and lateral facets of the trochlear groove; B) Trochlear depth is calculated as the average height of the medial (a) and lateral facet (b) minus the depth of the trochlear groove (c) [(a+b)/2-c]; C) Lateral trochlear inclination is the angle between a tangential line along the posterior femoral condyles (e) and the lateral facet of the trochlear groove (d); D) Femoral width is the maximum distance between the femoral epicondyles

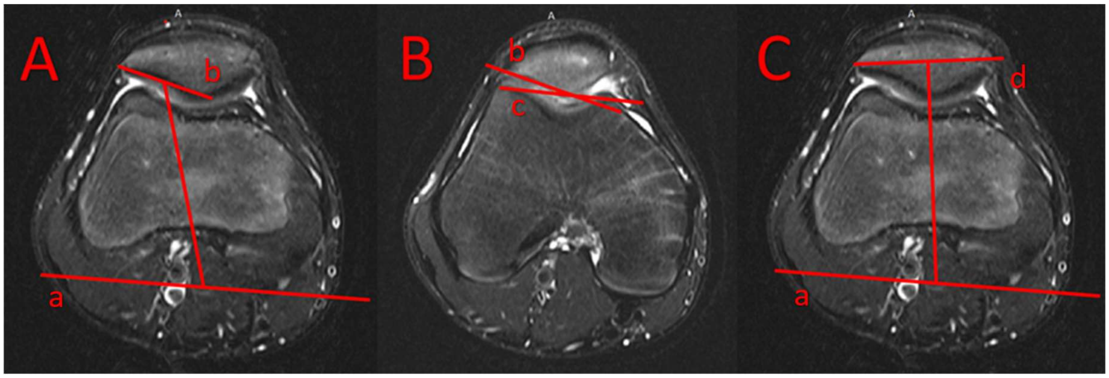


**Figure S2** A) The Fulkerson angle is defined as the angle between a tangential line along the posterior femoral condyles (a) and the lateral facet of the patella (b); B) The Laurin angle is measured between the anterior condyles (c) and the lateral patellar facet (b); C) The patellar tilt angle is the angle between a line along the posterior femoral condyles (a) and the largest axial diameter of the patella (d)

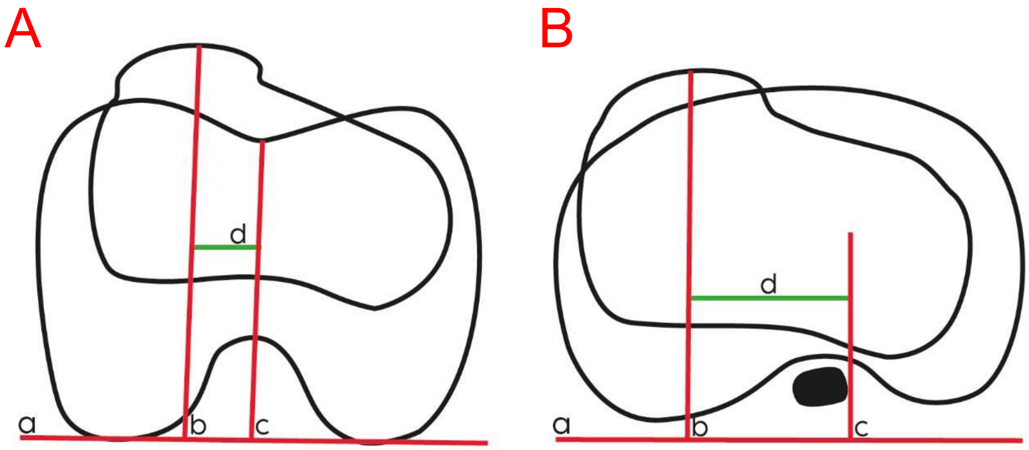


**Figure S3** A) The TTTG distance is measured as the distance (d) between two perpendicular lines drawn from a tangential line to the posterior condyles (a) to the trochlear groove (c) and the midpoint of the tibial tubercle (b); B) The TTPCL distance is determined by the distance (d) between two perpendicular lines extending from the posterior condyles (a) to the medial border of the PCL at its tibial insertion (c) and the midpoint of the tibial tubercle (b)

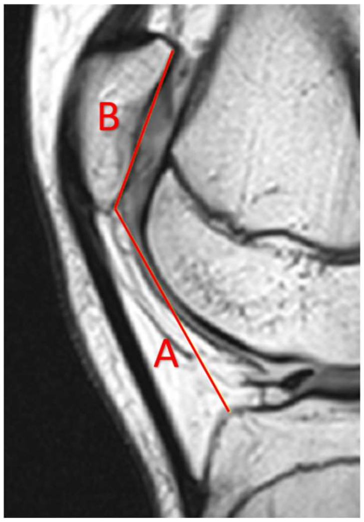


**Figure S4** The Caton-Deschamps Index (A/B) is the ratio of the distance from the anterior edge of the tibial plateau to the apex of the patella (A) divided by the length from the base to the apex of the patella (B)

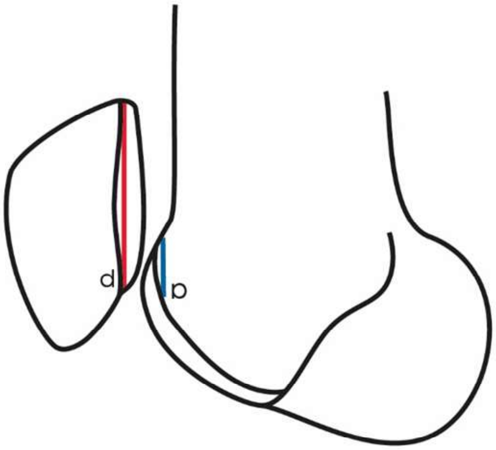


**Figure S5** The Patellotrochlear Index is the percentage of the femoral trochlea cartilage covering the cartilage of the Patella. The length of the cartilage of the femoral trochlea (a) is divided by the length of the cartilage of the Patella (b) multiplied by 100 in a sagittal view [(a/b)*100]; The sequence with the biggest diameter of the patella is to be picked for this measurement
